# Rapid transit in the left-sided colon is related to poor defecatory function at early period after stoma closure

**DOI:** 10.1038/s41598-020-60808-7

**Published:** 2020-02-28

**Authors:** Ri Na Yoo, Hyeon-Min Cho, Bong-Hyeon Kye, Hyung Jin Kim, Sukhyun Shin, Gun Kim

**Affiliations:** Division of Colorectal Surgery, Department of Surgery, St. Vincent’s Hospital, College of Medicine, The Catholic University of Korea, Suwon, Gyeonggi-do Korea

**Keywords:** Enteric neuropathies, Rectal cancer

## Abstract

Sphincter-saving surgery (SSS) is the gold standard for rectal cancer surgery but results in a wide spectrum of bowel dysfunction. This study investigated the impact of colonic dysmotility on the incontinent form of bowel dysfunction. Bowel function of patients who received SSS with loop ileostomy for treating rectal cancer was reviewed retrospectively from June 2013 two August 2015 at a single hospital. Immediately after closure of a diverting stoma, patients were tested for the colonic transit time (CTT) using radiopaque markers. Bowel dysfunction at 6 and 12 months after SSS was measured as the severity of fecal incontinence according to the Cleveland Clinic Incontinence Score (CCIS) and the use of an anti-diarrheal drug. A short CTT for the left colonic segment was significantly associated with the high CCIS and use of an antidiarrheal agent at 6 months after sphincter preservation. However, the CTT didn’t correlate with the CCIS at 12 months after SSS. Rather, age and surgical method demonstrated a significant association. Colonic dysmotility after SSS appears to intensify fecal incontinence for a relatively short period. Its impact abates within a year.

## Introduction

With the advent of modern surgical techniques and chemoradiation therapy, sphincter-saving surgery (SSS) has become the gold standard treatment in patients with rectal cancer^1–3^. As the survival outcome improves, issues on quality of life for cancer survivors are now greatly emphasized. Up to 80% of patients undergoing SSS reported a broad spectrum of significant bowel dysfunction postoperatively, defined as low anterior resection syndrome (LARS)^4^. Because LARS perniciously affects daily life, the quality of life of patients who have undergone SSS cannot be deemed superior to that of patients with permanent colostomy^5,6^.

The demand to improve quality of life of patients with bowel dysfunction is increasing. However, current treatments for LARS are merely empirical, controlling symptoms of incontinence, frequent diarrhea, and constipation^7^. Multifactorial etiologies and the complex, intertwined pathophysiology of LARS render diagnosis and treatment difficult. Some risk factors for LARS are known, such as an alteration in anorectal physiology, injury to the anal sphincter or pudendal nerve, and colonic dysmotility. Nevertheless, most of the mechanisms and much of the pathophysiology of LARS remain to be elucidated^8,9^. From daily incontinence with urgency and soiling to constipation and obstructive defecation, patients present with a wide spectrum of bowel dysfunction after rectal resection. This study aims to evaluate the impact of colonic dysmotility on incontinent symptoms of LARS through a colonic transit time (CTT) test on patients with multiple risk factors for LARS.

## Result

**Figure 1:**
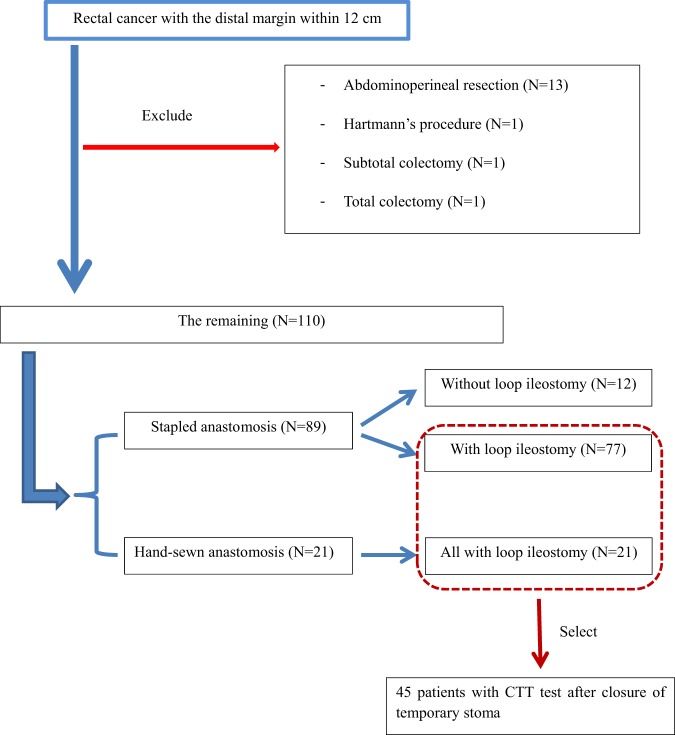
Inclusion and exclusion criteria. Inclusion and exclusion criteria are depicted above.

A total of 126 patients with rectal cancer in which the distal margin was within 12 cm from the anal verge underwent radical rectal resection during the designated period. Excluding patients who underwent abdominoperineal resection, Hartmann’s procedure, subtotal colectomy, or total colectomy, 110 patients were identified to have undergone SSS (12 patients without loop ileostomy and 98 with loop ileostomy). Among those with loop ileostomy, the CTT test was conducted in 45 patients (Fig. 1).

Table 1 lists the demographics of the patients in this study. The mean age was 62.9 years, ranging from 38 to 75 years. The sex ratio was 25 males to 20 females. Of 45 patients, 36 underwent low anterior resection with stapled anastomosis. The other nine patients received intersphincteric resection (ISR) with hand-sewn anastomosis. The mean distance of the distal margin of the tumor from the anal verge was 7.8 cm. The average CCIS was 10.3 at 6 months and 8.9 at 12 months. The average defecation frequency per day was 8.6 times at 6 months and 7.7 at 12 months. The mean CTT immediately after the closure of a diverting stoma was 52.5 hours for the entire remnant colon, 43.8 hours for the right, 7.2 hours for the left, and 1.5 hours for the rectosigmoid.

**Table 1:** Demographics.

		Range	
Age (years)	**62.9 ± 9.6**	38~75	
Sex (male:female)	**25:20**		
Operation method (SA: CAA)	**36:9**		
Tumor location (cm from anal verge)	**7.8 ± 2.4**	2.7~12.0	
Defecation			
CCIS at 6months	**10.3 ± 4.9**	0~20	
CCIS at 12months	**8.9 ± 4.6**	0~20	
Defecation/day at 6months	**8.6 ± 4.3**	2~20	
Defecation/day at 12months	**7.7 ± 5.2**	2~30	
Colon transit time			Reference value^13^
Total Transit time (hours)	**52.5 ± 27.6**	10.0~135.8	20.5~30.1
Right (hours)	**43.8 ± 24.6**	7.4~121.4	5.4~6.8
Left (hours)	**7.2 ± 6.3**	0.2~24.4	10.8~12.5
Recto-sigmoid (hours)	**1.5 ± 1.9**	0.0~9.4	5.7~7.7

CCIS, Cleveland Clinic Incontinence Score SA, Stapled anastomosis, CAA colo-anal anastomosis.

After patients with a CCIS greater than 9 were compared with those having a score equal to or less than 9 at 6 months after SSS, the CTT for the left side of the remnant colon was significantly shorter in patients with a CCIS greater than 9 than in the others, as shown in Table 2. Furthermore, with marginal significance, patients with a CCIS greater than 9 tended to be older than those with a CCIS equal to or less than 9. The tumor location was closer to the anal verge in patients with a CCIS greater than 9 with marginal significance. When patients who used an antidiarrheal agent at 6 months after SSS were compared with those who did not, the CTT for the left and rectosigmoid area was significantly shorter, as shown in Table 3. Patients who used an antidiarrheal agent were older than those without such use.

**Table 2:** Cleveland Clinic Incontinence Score at 6 months after sphincter-saving surgery.

	CCIS ≤ 9 (N = 19)	CCIS > 9 (N = 26)	*P*-value	
			Uni-variate	Multi-variate
Age (years)	59.9 ± 9.9	65.1 ± 8.8	0.078	0.193
Sex				
Male	13 (52.0%)	12 (48.0%)	0.224	
Female	6 (30.0%)	14 (70.0%)		
Tumor location (cm from AV)	8.6 ± 1.8	7.2 ± 2.6	0.063	0.065
OP method				
SA	16 (44.4%)	20 (55.6%)	0.712	
CAA	3 (33.3%)	6 (66.7%)		
**Colon transit time**				
Total (hours)	55.9 ± 24.8	47.9 ± 31.2	0.346	
Right (hours)	45.6 ± 22.5	41.5 ± 27.6	0.585	
Left (hours)	8.9 ± 7.1	4.7 ± 4.2	**0.026**	**0.021**
Recto-Sigmoid (hours)	1.4 ± 1.6	1.7 ± 2.4	0.554	

CCIS, Cleveland Clinic Incontinence Score SA, Stapled anastomosis, CAA colo-anal anastomosis.

**Table 3:** The use of anti-diarrheal agent at 6 months after sphincter-saving surgery.

	LOP (−) (N = 17)	LOP (+) (N = 28)	*P*-value	
			Uni-variate	Multi-variate
Age (years)	59.6 ± 11.9	64.9 ± 7.4	0.076	0.125
Sex				
Male	8 (32.0%)	17 (68.0%)		
Female	9 (45.0%)	11 (55.0%)	0.537	
Tumor location (cm from AV)	8.5 ± 1.9	7.3 ± 2.5	0.13	
OP method				
SA	14 (38.9%)	22 (61.1%)		
CAA	3 (33.3%)	6 (66.7%)	0.757	
Colon transit time				
Total (hours)	55.7 ± 31.8	50.6 ± 25.2	0.547	
Right (hours)	43.7 ± 27.9	43.9 ± 22.8	0.981	
Left (hours)	9.6 ± 7.7	5.7 ± 4.8	**0.040**	0.103
Recto-sigmoid (hours)	2.4 ± 2.7	1.1 ± 1.1	**0.017**	0.137

CCIS, Cleveland Clinic Incontinence Score SA; Stapled anastomosis; CAA colo-anal anastomosis; LOP, loperamide.

As listed in Table 4, patients with a CCIS greater than 9 at 12 months were significantly older than the others. Moreover, they exhibited a tumor located lower in the rectum and received ISR more frequently. However, the CTT was not significantly different between the two groups. Patients who used an antidiarrheal agent at 12 months exhibited similar characteristics: older age and the tumor being located lower in the rectum, as shown in Table 5. The patients who underwent ISR tended to use an antidiarrheal agent at 12 months more frequently than the others did.

**Table 4:** Cleveland Clinic Incontinence Score at 12 months after sphincter-saving surgery.

	CCIS ≤ 9 (N = 27)	CCIS > 9 (N = 18)	*P*-value	
			Uni-variate	Multi-variate
Age (years)	60.5 ± 10.7	66.5 ± 6.1	**0.037**	**0.038**
Sex				
Male	14 (56.0%)	11 (44.0%)		
Female	13 (65.0%)	7 (35.0%)	0.76	
Tumor location (cm from AV)	8.4 ± 2.0	6.8 ± 2.7	**0.032**	0.883
OP method				
SA	25 (69.4%)	11 (30.6%)		
CAA	2 (22.2%)	7 (77.8%)	**0.019**	0.053
**Colon transit time**				
Total (hours)	57.7 ± 29.6	44.8 ± 22.9	0.126	
Right (hours)	47.2 ± 26.5	38.7 ± 21.1	0.259	
Left (hours)	8.5 ± 6.5	5.0 ± 5.4	0.069	0.191
Recto-sigmoid (hours)	1.9 ± 2.2	0.9 ± 1.4	0.123	

CCIS, Cleveland Clinic Incontinence Score SA, Stapled anastomosis, CAA colo-anal anastomosis.

**Table 5:** The use of anti-diarrheal agent at 12 months after sphincter-saving surgery.

	LOP (−) (N = 15)	LOP (+) (N = 30)	*P*-value	
			Uni-variate	Multi-variate
Age (years)	56.5 ± 11.4	66.1 ± 6.7	**0.001**	**0.005**
Sex				
Male	9 (36.0%)	16 (64.0%)		
Female	6 (30.0%)	14 (70.0%)	0.757	
Tumor location (cm from AV)	8.8 ± 1.5	7.3 ± 2.6	**0.047**	0.499
OP method				
SA	14 (38.9%)	22 (61.1%)		
CAA	1 (11.1%)	8 (88.9%)	0.089	0.426
Colon transit time				
Total (hours)	57.4 ± 33.2	50.1 ± 24.6	0.409	
Right (hours)	48.1 ± 29.1	41.7 ± 22.2	0.421	
Left (hours)	7.5 ± 6.1	7.1 ± 6.5	0.828	
Recto-sigmoid (hours)	1.9 ± 1.7	1.4 ± 2.1	0.384	

CCIS, Cleveland Clinic Incontinence Score SA; Stapled anastomosis; CAA colo-anal anastomosis; LOP, loperamide.

## Discussion

Investigating the impact of colonic dysmotility on the symptoms of LARS, this study demonstrated that the colonic motility of the mobilized, left colon seems to be correlated with the symptom of fecal incontinence, as reflected by the CCIS at postoperative 6 months. Such a consequence is partially explained by the denervation of the left colon during transection of the IMA at its origin and mobilization of the left colon^9,10^. Previously, a canine model of the anterior resection state demonstrated that injuries to the descending fibers of the inferior mesenteric plexus and to the ascending fibers of the pelvic plexus seem to shorten the CTT for the mobilized colonic segment mediated by dominant inhibitory sympathetic pathways in the distal colon^11^. Although the level of arterial ligation in rectal cancer surgery is still controversial, high ligation of the IMA gives advantages in regards to oncologic surgery, such as en block dissection of the lymph node, low level of anastomosis without tension, and the precise tumor staging^12^. However, it is inevitable to sacrifice some descending fibers of the IMA plexus to perform the high ligation of IMA. The present study also demonstrated that the denervation around the origin of the IMA resulted a shorter CCT, compared with the reference value for healthy Korean adults^13^.

By correlating the CCIS with CTT test result obtained during the postoperative period of ileostomy closure, this study showed that the CTT is associated with the degree of fecal incontinence and use of an antidiarrheal agent at postoperative 6 months from index surgery. In other words, a shorter CTT for the left and neorectosigmoid colon is associated with a higher CCIS and greater use of an antidiarrheal agent. At postoperative 12 months, the CTT for the left and neo-rectosigmoid colon for patients with a CCIS greater than 9 is shorter than that for patients with a CCIS less than or equal to 9. However, the CTT value at 12 months was not statistically significant. By contrast, old age and ISR along with a low tumor location are correlated with incontinent symptoms at postoperative 12 months. This suggests that a rapid colonic transit time in an early following period is alleviated within a year.

The nerve innervation may explain the recovery of colon motility. The newly constructed left side of the colon is mainly distal transverse colon and proximal descending colon, which is innervated by not only from the IMA but also SMA plexus. Colonic motility would be influenced primarily by the left colon postoperatively because the right colon is not affected by the nerve injuries around the IMA plexus. The neo-rectosigmoid colon would likely function as a passage rather than a functional reservoir since pelvic fibrosis and injury to the IMA plexus probably induce the complete loss of bowel motility in the neo-rectosigmoid colon. The left colon seems to recover the colonic motility in a year, and the recovery possibly involves the effect of SMA plexus.

Aging, the capacity of the remnant rectum, and iatrogenic sphincter injury during index surgery are more potent factors for LARS than colonic dysmotility caused by nerve injury during rectal resection. In cases of patients with multiple risk factors for a major LARS, including undergoing high ligation of IMA, use of neoadjuvant chemoradiation therapy, use of temporary stoma, and very low anastomosis, the alteration in anorectal physiology shortly after the rectal resection may benefit from an active intervention, such as using antidiarrheal agent.

There are several limitations to this study. First, the effect of ileostomy was not well addressed because all study patients underwent loop ileostomy simultaneously at the time of rectal resection. It is suggested that by causing disuse atrophic colitis, loop ileostomy plays a critical role in the development of major LARS^14,15^. A previous study on epithelial cell kinetics in the human rectal mucosa showed that on reversal of bowel continuity, the rectal mucosa regained intestinal musculature and normal mucosal function^16^. A recent study reporting the factors related to major LARS demonstrated that only neoadjuvant chemoradiation therapy and colo-anal anastomosis were significantly associated with the development of major LARS^17^. However, a risk factor analysis of LARS revealed that patients who once had loop ileostomy around the time of rectal resection persistently experienced bowel dysfunction for a prolonged period of up to 5 years^15^. Although several risk factors related to the development of LARS are speculated, the pathogenesis of how independent factors provoke the alteration of anorectal physiology remains to be clarified. Because the study population was evaluated under same condition, it is reasonable to hypothesize that each risk factor for LARS minimally influenced the study result. Postoperative ileus and the use of narcotics can be problematic for bowel motility postoperatively. According to Vather *et al*., postoperative ileus is the presence of at least two of the following signs or symptoms on or after postoperative day 4: nausea and vomiting, inability to tolerate a solid or semiliquid diet during the preceding 24 hours, abdominal distension, and radiological evidence of ileus^18^. Because all patients in this study underwent radiological evaluation for bowel motility, postoperative ileus could be disregarded. In addition, the use of narcotics was not limited during the early postoperative period. However, once patients started on oral feeding, only an oral form of NSAIDs was administered for pain control; therefore, the effect of narcotics on bowel motility would be minimal. Finally, the retrospective nature and small sample size of the study cannot be disregarded. Despite these limitations, and to the best of our knowledge, this study is the first to objectively measure the colonic dysmotility associated with LARS in order to delineate the possible pathophysiology of LARS.

## Conclusion

Using the CTT for the left colon at the time of ileostomy closure, colonic dysmotility can be measured. By correlating the CTT with the CCIS, colonic dysmotility is observed within a short period following SSS. However, the influence of the CTT on LARS diminishes over time, whereas age and tumor height are markedly more influential in determining the level of anastomosis and accordant surgical approach to LARS.

## Method

Patients who underwent radical rectal resection for clinical stage II and III rectal cancer located within 12 cm of the anal verge between June 2013 and August 2015 were retrospectively analyzed. Patients who received abdominoperineal resection, Hartmann’s procedure, subtotal colectomy, or total colectomy were excluded. On patients with loop ileostomy and without the evidence of anastomotic leakage, the CTT test was conducted after closure of a diverting stoma. Patients with a CTT test result were included in this study. This study was approved by the Institutional Review Board of St. Vincent’s Hospital, the Catholic University of Korea (IRB No.VC16RISI0216). The documentation of informed consent was waived by the IRB because of the retrospective nature of the study and the analysis used anonymous clinical data. Also, this study did not involve any additional procedure other than the routine practice given in clinical setting, presenting no more than minimal risk of harm to subjects.

All patients included in this study received preoperative long-course chemoradiation therapy within 6 to 8 weeks before SSS. A 5-fluorouracil-based regimen was administered concurrently to the pelvis with radiation of 45 to 50 Gy in 25 to 28 fractions. For the surgical procedure, high ligation of the inferior mesenteric artery (IMA) and mobilization of the splenic flexure were performed routinely. Total mesorectal excision was carried out as a standard procedure. To secure the integrity of anastomosis, temporary loop ileostomy was performed. The closure of a diverting stoma was performed 12 to 14 weeks after SSS. The wait period for the closure of a diverting stoma was based on the schedule for the adjuvant chemotherapy given during the following six months. All patients received the 5-fluorouracil-based regimen given every 4 week. The temporary stoma was closed just before the last cycle of the adjuvant chemotherapy.

Retrospective analysis was conducted after data were prospectively collected on patients’ demographics, operation type, tumor location, and information on defecation, including the frequency, use of an antidiarrheal drug, and Cleveland Clinic Incontinence Score (CCIS) gathered at postoperative 6 and 12 months after SSS.

All patients followed a standardized clinical pathway after closure of loop ileostomy. On postoperative day 3, oral hydration with clear water was allowed. On postoperative day 4, a liquid diet was started, and on day 5, a soft diet. The CTT test was started on postoperative day 4. Three capsules of KolomarkTM (M.I. Tech, Pyeongtaek, South Korea), containing 20 radiopaque markers in each capsule, were administered with water after the first liquid meal in the morning. From day 4, a kidney–ureter–bladder X-ray was taken twice daily, morning and evening, until the radiopaque markers disappeared. During this period, patients were not administered laxatives or other medications that might affect bowel motility.

The CTT was calculated based on an equation modified from Arhan’s theory 10. Thus, the CTT was calculated from X-rays taken every 24 hours after a single dose of 20 radiopaque markers was administered. The equation below, on the left side, was used: Because our study patients generally presented with rapid transit after rectal resection, a single dose of radiopaque markers was washed out within a day, hindering a precise measure of the transit time. Thus, in this study, all patients were administered a triplet of 20 markers, and an X-ray was taken twice daily. By considering the use of 60 radiopaque markers and an X-ray taken every 12 hours, the CTT was calculated using the modified equation written below on the right side.

$${\rm{CTT}}=1.2{\sum }_{n=1}ni\,\to \,{\rm{CTT}}=\frac{1.2}{3\,\ast \,2}{\sum }_{i=1}ni$$:

n = the total number of markers present in each dose: I = total number of films

**Figure 2:**
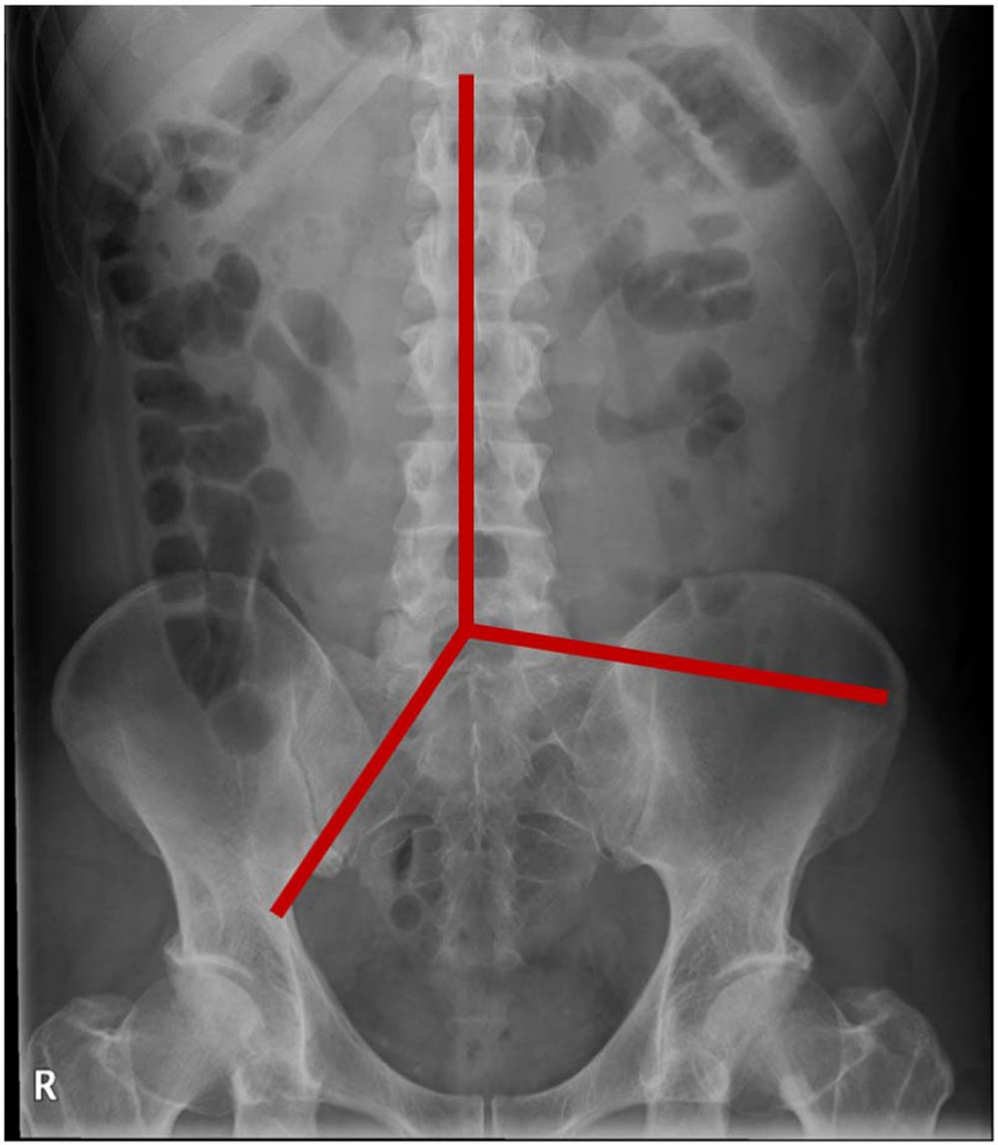
Three colonic segments. The colonic segments are divided by the landmarks of the spinal process and imaginary lines from the fifth lumbar vertebra to the right pelvic outlet and left iliac crest.

The remaining colonic segment after SSS was divided into three sections: right, left, and rectosigmoid, based on the landmarks of the spinal process and imaginary lines from the fifth lumbar vertebra to the right pelvic outlet and left iliac crest, as shown in Fig. 2 ^19,20^. The CTTs for the right, left, rectosigmoid, and total colon were calculated.

Patients were divided into two groups according to the CCIS at 6 and 12 months: one with a CCIS equal to or less than 9 and another with a CCIS greater than 9. Both groups were compared to determine differences in demographic factors and CTT. In addition, according to the use of an antidiarrheal agent at 6 and 12 months, patients were divided into two groups: one without the use of such an agent and the other with the use. Both groups were compared in the same manner as well.

Continuous variables were analyzed with Student’s t test. Categorical variables were analyzed using a chi-squared test. A logistic regression model was used to compare the factors considered significant in univariate analysis. Statistical significance was defined as p ≤ 0.05. A p value, greater than 0.05 but less than 0.1, was considered marginally significant. Statistical calculation was performed using SPSS 15.0 for Windows (SPSS, Inc., Chicago, IL, USA).
